# The role of the PI3K/Akt/mTOR signalling pathway in human cancers induced by infection with human papillomaviruses

**DOI:** 10.1186/s12943-015-0361-x

**Published:** 2015-04-17

**Authors:** Lifang Zhang, Jianhong Wu, Ming Tat Ling, Liang Zhao, Kong-Nan Zhao

**Affiliations:** Institute of Molecular Virology and Immunology, Wenzhou Medical University, Wenzhou, 325035 Zhejiang PR China; Australian Prostate Cancer Research Centre-Queensland, Institute of Health and Biomedical Innovation, Queensland University of Technology, 37 Kent Street, Woolloongabba, Brisbane, 4102 QLD Australia; The University of Queensland, Brisbane, 4072 QLD Australia; Centre for Kidney Disease Research-Venomics Research, The University of Queensland School of Medicine, Translational Research Institute, 37 Kent Street, Woolloongabba, Brisbane, 4102 QLD Australia; Current address: Department of Gastric Cancer and Soft Tissue Sarcomas Surgery, Fudan University Shanghai Cancer Center, Shanghai, 200032 PR China

**Keywords:** Cancer, Human papillomavirus, E6, E7, E5, Phosphatidylinositol 3-kinase (PI3K), Akt, Mammalian target of rapamycin (mTOR)

## Abstract

Infection with Human papillomaviruses (HPVs) leads to the development of a wide-range of cancers, accounting for 5% of all human cancers. A prominent example is cervical cancer, one of the leading causes of cancer death in women worldwide. It has been well established that tumor development and progression induced by HPV infection is driven by the sustained expression of two oncogenes E6 and E7. The expression of E6 and E7 not only inhibits the tumor suppressors p53 and Rb, but also alters additional signalling pathways that may be equally important for transformation. Among these pathways, the phosphatidylinositol 3-kinase (PI3K)/Akt/mammalian target of rapamycin (mTOR) signalling cascade plays a very important role in HPV-induced carcinogenesis by acting through multiple cellular and molecular events. In this review, we summarize the frequent amplification of PI3K/Akt/mTOR signals in HPV-induced cancers and discuss how HPV oncogenes E6/E7/E5 activate the PI3K/Akt/mTOR signalling pathway to modulate tumor initiation and progression and affect patient outcome. Improvement of our understanding of the mechanism by which the PI3K/Akt/mTOR signalling pathway contributes to the immortalization and carcinogenesis of HPV-transduced cells will assist in devising novel strategies for preventing and treating HPV-induced cancers.

## Introduction

Human papillomaviruses (HPVs) are non-enveloped, epitheliotropic, circular double-stranded DNA viruses [1,2]. HPV infection leads to many different cancers [1,3-5]. It has been well established that high-risk sexually transmitted HPVs such as HPV 16, 18, 31, 33, 35, 39, 45, 51, 52, 56, 58, 59, 68, 73, and 82 may lead to the development of cervical intraepithelial neoplasia (CIN), vulvar intraepithelial neoplasia (VIN), penile intraepithelial neoplasia (PIN), and anal intraepithelial neoplasia and squamous cell carcinoma (AIN) while cutaneous β-HPVs such as HPV 2, 4, 5, 8, 10 and 15 are suspected to have an etiologic role in skin warts and cancers [3,6-8]. In total, an estimated 5% of human cancers are caused by HPV infections [9].

Genomic instability is a hallmark of carcinogenesis and recognised as an important factor in the accumulation of mutated genes required for carcinogenesis [10]. Endogenous mutations and accumulation of mutational events are very important in the pathogenesis of premalignant lesions and tumour progression, which promote genomic instability to decrease the ability of maintaining the fidelity of DNA sequences [3,11,12]. Published studies have shown that HPV infection causes genomic instability (chromosomal gain or loss) and gene alterations including endogenous mutations and increased DNA damage which are associated with cancer development [13,14]. In HPV-positive cervical and vulva squamous cell carcinomas, the most common lesions were the loss of 11q and gains of 3q, the latter has been found in more than 25% of high grade CIN [13]. A more comprehensive understanding of genomic instability and mutational events associated with the development of cancers caused by HPV infection is needed and will be separately discussed.

It has been well established that HPV E6 and E7 oncogenes inactivate two tumor suppressors (p53 and pRb) in virus-infected cells. Molecular and cell biology approaches have revealed that alterations of additional signalling pathways are equally important for transformation of HPV oncogene-transduced cells [15]. It is now widely accepted that PI3K/Akt/mTOR signalling pathway plays a pivotal role in many human cancers. HPV infection accompanied by E6/E7 expression activates this signalling pathway by altering multiple cellular and molecular events to drive carcinogenesis [16-18]. The PI3K pathway is unique, in that all of the major components of this pathway have been found to be frequently amplified or mutated in HPV-induced cancers [19-24]. The PI3K/Akt/mTOR signalling pathway mediates the multiple cellular and molecular functions through the altered expression of its targeted genes, which are critical to tumor initiation, progression and outcomes [25,26]. Thus, this pathway has been proposed as a promising therapeutic target for many cancers including cervical cancer [27]. In this review, we summarise the current knowledge of the roles of the PI3K/Akt/mTOR signalling pathway in HPV-induced cancers.

## HPV life cycle and ATM /p38MAPK/MK2 pathways

HPVs whether they are low-risk and high-risk are epitheliotropic. Infection with two HPVs may be latent or active [28]. The latent HPV infection will complete the viral life cycle to produce virus progeny, which arises via the distinctly different mechanisms from those involved in active HPV infection [29]. During the HPV life cycle, genome amplification is necessary for production of the virus progeny that is prevented until the levels of viral replication proteins rise, and depends on the co-expression of several viral proteins [30]. Expression of E6 and E7 in the lower epithelial layers drives cells into S-phase creates an environment that is conducive for viral genome replication and cell proliferation [31,32]. The lower epithelial layers where HPVs can establish their infection are the only compartment to contain the cells progressing through the cell cycle [33]. Viral capsid proteins (L1 and L2) are expressed to assemble the virus progeny in cells upon their differentiation that also express E4 to complete its life cycle when the infected cell enters the upper epithelial layers [29]. We have confirmed that expression of HPV6b and BPV1 L1 proteins is dependent on cell differentiation in primary keratinocyte culture systems [34-37]. Thus, the late phase of HPV life cycle is closely linked to the differentiation state of the stratified epithelium it infects, with progeny virus only made in the terminally differentiating suprabasal compartment [38].

It has been established that the cellular DNA damage response (DDR) is activated during the HPV life cycle [39]. This activation leads to the induction of an Ataxia-telangiectasia mutation (ATM)-dependent signalling cascade, DNA repair and cell cycle arrest during G2/M to avoid further DNA damage [15,30,34,38,40-42]. Thus, G1, S, G2, and early M phase cell cycle inhibitors efficiently prevented the virus infection [33]. The ATM pathway is responsible for the DDR to double-strand DNA breaks, which is mediated through the action of downstream kinases, such as CHK2 [39,42,43]. The E1 gene might play a key role in this process, which causes double-strand DNA breaks in the host genome [40,44]. By activating the ATM pathway, HPV recruits cellular DNA repair and recombination factors into its replication centers during the stable and vegetative phases of its life cycle [45]*.* In cells with impaired p53 activity, DNA damage repair requires the activation of p38MAPK along with MAPKAP kinase 2 (MK2) [43]. In HPV-positive cells, phosphorylation of p38 and MK2 proteins was induced along with relocalization to the cytoplasm. Treatment with MK2 or p38 inhibitors blocked HPV genome amplification, confirming the p38/MK2 pathway as a key regulator of the HPV life cycle [43]. Thus, it appears to be clear that the ATM/p38MAPK/MK2 pathways are required for HPVs to complete normal life cycle in the host body.

## HPV infection, carcinogensis and PI3K/Akt/mTOR signalling pathway

**Figure 1:**
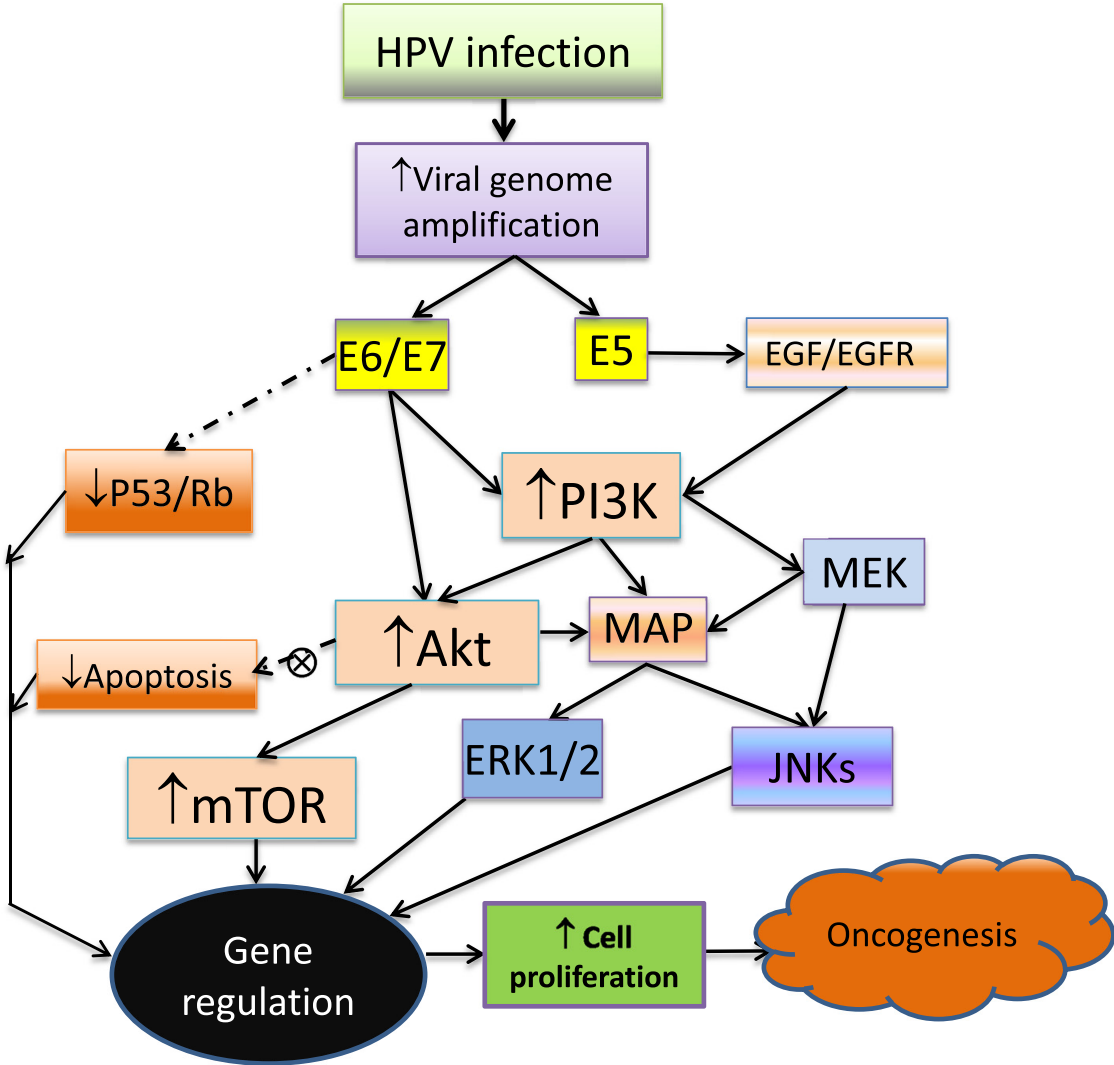
HPVs infect host epithelial cells (keratinocytes) by interacting with different cell surface receptors such as integrin and heparan sulfate proteoglycans (HSPGs). The HPVs replicate themselves using the host cell replication apparatus to express E6/E7/E5 oncoproteins to immortalize the infected cells not only by inhibiting tumour suppressors p53 and Rb and decreasing apoptosis, but also importantly by activating the PI3K/Akt/mTOR signalling pathway. All these processes enhance cell proliferation leading to the carcinogenesis. Solid line: stimulatory influence; Dashed line: inhibitory influence; ⊗: defective process.

Active HPV infection which is also known as abortive infection leads to induction of cancer including benign and malignant neoplasms [46]. In the case of carcinogenesis, viral infection induces the initiation and development of cervical and other cancers via their interactions with different cellular signalling pathways in host cells [47]. In addition to the inhibition of p53 and pRb, HPVs also interact with four major upstream pathways (growth factor receptor, notch receptor, *Ras* and *PI3KCA* genes) to stimulate host cell survival and proliferation, leading to carcinogenesis through activation and alteration of the components of the PI3K/Akt/mTOR pathway [19,48-53] (Figure 1).

### PI3K

PI3K modulates different signals to prevent apoptosis and promote cellular survival and proliferation in a wide variety of cell types [54,55]. It has been shown that PI3K is amplified and activated in HPV-induced cervical cancers and other cancers [56]. For instance, PI3K activity is significantly increased in laryngeal papilloma (a hyperplastic tumor of the respiratory tract induced by HPV 6/11), leading to upregulation of EGFR and subsequently activation of MAPK/ERK [57,58]. The activation of MAPK/ERK in turn alters transcription of multiple genes that are important for cell cycle regulation and cell proliferation. Furthermore, HPV infection causes laryngeal epithelial cells to develop recurrent respiratory papillomas where expression of keratin-13 (K13), a cell differentiation marker of human mucosal keratinocytes, is diminished [58]. This is due to the increased PI3K activity that enhances papillomas cell proliferation and represses terminal differentiation (and hence the failure to upregulate K13 expression) [58].

Liu et al. reported that BPV-1 L1 and L2 pre-mRNAs are spliced in keratinocytes, which contain two exonic splicing enhancers [59]. Each exonic splicing enhancer has an alternative splicing factor (ASF) and splicing factor 2 (SF2), which together play an important role in viral RNA expression and splicing at the proximal 3′ splice site [59]. Depletion of ASF/SF2 from the cells greatly decreases viral RNA expression and RNA splicing at the proximal 3′ splice site. Activation-rescued viral RNA expression and splicing in ASF/SF2-depleted cells are mediated through the PI3K/Akt pathway and associated with the enhanced expression of other serine/arginine-rich (SR) proteins [59]. The ASF/SF2 co-operate with H-Ras to enhance cellular proliferation and protect cells from apoptosis by upregulating expression of phosphorylated SR proteins (SRp30s and SRp40) through the PI3K/Akt pathway in cervical and other cancers [59,60]. A published study has also reported that HPV infection-induced IL-17 expression can stimulate Mcl-1 expression to promote lung tumor cell progression through the PI3K pathway [61].

### Akt

Akt is a serine/threonine-specific protein kinase, which plays a key role in multiple cellular processes including apoptosis and cell proliferation. Phosphorylation and activation of Akt also play an important role in the majority of HPV-caused malignancies including anal squamous cell carcinomas (ASCCs) [7]. Sixty six percent (82/125) of ASCCs show cellular accumulation of p-Akt associated with nuclear accumulation of MDM2 [7]. Thirty nine out of 46 formalin-fixed cervical neoplastic specimens showed p-Akt on serine 473 [62]. Forty-eight percent (12/25) of cervical cancer patients at stage Ib2-IIb exhibited Akt activation in cancer cells [63]. The radiation sensitivity of HPV-infected H&N cancers also correlates to Akt activation [64]. Mechanistically, HPV infection causes genome instability due to integration of the viral DNA into the host genome. Thus, mutations of PIK3CA gene (which encodes PI3K) in HPV-infected cells and tissues or HPV-DNA transformants may account for Akt activation present in cervical and other types of HPV-induced cancers, since PIK3CA shows the highest frequency of gain-of-function mutations in these cancers [20,63,65]. Oncogenic mutations and amplification of PIK3CA activate PI3K/Akt signalings to drive the HPV-induced tumorigenesis [19,21,65].

Akt phosphorylation is required for a BRCT (BRCA1 carboxyl-terminal) domain-containing protein TopBP1 to interact with other transcription factors, especially E2F1. E2F1 functions as a tumor suppressor to induce apoptosis [66,67]. Phosphorylated Akt (p-Akt) interacts with E2F1 to repress its proapoptotic activity and induce oligomerization of TopBP1 [66]. Furthermore, an endosomal/lysosomal cysteine protease cathepsin L (Ctsl) has been reported to act as an anti-tumor protease. Ctsl is critical for termination of growth factor signalling in the endosomal/lysosomal compartment of keratinocytes and has an inhibitory role in Akt activation in HPV-induced carcinogenesis [68,69]. Therefore, activation of Akt/MAPK pathway is only upregulated in Ctsl-deficient cells [68]. Additionally, papillomavirus-binding factor (PBF), a transcriptional regulator, controls the promoter activity of HPVs by binding to the regulatory sequences of certain papillomavirus types and Huntington’s disease binding protein 2 (HDBP2) through the 14-3-3β protein via two motifs (RSRSLSFSEP and LSKSAP) [70,71]. Activated Akt phosphorylates the two motifs, allowing PBF to associate with 14-3-3β to promote cell survival and growth [70]. These studies suggest that activation of Akt may contribute to the HPV-induced tumorigenesis. It has been reported that high levels of p-Akt might be an unfavourable prognostic marker for relapse-free survival in oropharyngeal cancer [51].

Mammalian genomes contain three Akt genes, Akt1, Akt2 and Akt3. Akt1 encodes the principal Akt isoform that regulates apoptosis [72]. HPVs may differentially affect epithelial Akt activity, as the three Akt isoforms behave differently during epidermal tumorigenesis [73,74] . Cutaneous HPV8 early genes reduce epidermal Akt activity primarily due to down-regulation of Akt1. In contrast, Akt activity can be focally stimulated by up-regulation and phosphorylation of Akt2 in both papillomas and HPV gene-induced epidermal tumours. In SCC, Akt1 is commonly down-regulated consistent with a viral influence, whereas Akt2 up-regulation is widespread. Activation of upregulated Akt2 by serine phosphorylation associates with high-grade tumours, and is characteristic of SCC associated with malignancy [74]. Interestingly, high level of Akt2 is often associated with the presence of β-HPV species (HPV 15) and the up-regulation of p16INK4a and Akt/PI3K pathways [51]. p-Akt2 is over-expressed in basal cell carcinoma (BCC) accompanied by up-regulation of tumor suppressor p16INK4a [51,75]. Overexpression of p16INK4a is common in cervical cancer where pRb protein is inactivated by high-risk HPVs. However, it is still unclear whether p16INK4a overexpression can be used as an indicator of pathogenic activity of high-risk HPVs. Nevertheless, the PI3K/ Akt /mTOR pathway is associated with the up-regulation of p16INK4a by HPVs [19,65,75-79]. So far, it remains unclear whether and how Akt 3 plays a functional role in HPV-induced tumorigenesis.

### mTOR

mTOR kinase acts as a cellular rheostat that integrates the signals from a variety of cellular signalling pathways to sense growth factor, nutrient availability and energy status. Recently, it has been reported that activation of Akt /mTOR can be detected within several minutes following exposure of human keratinocytes to HPV16 pseudovirions [80]. mTOR activation is frequently observed in cervical squamous cell carcinoma, most HPV(+) head and neck squamous cell carcinomas (HNSCC), HPV(+) oropharyngeal cancers (OPSCC), cervical cancer squamous cell carcinomas (CCSCC) lesions and cell lines [2,17,63,81]. A tissue microarray analysis has shown that 13 cervical cancer patients (52%) express phosphorylated mTOR (p-mTOR) in the cytoplasm and membrane of cancer cells [63]. Both p-mTOR expression and distant metastasis significantly correlate with the response to nucleus accumbens core [63]. Another analysis of 20 samples each of normal cervix, high-grade squamous intraepithelial lesions (HSIL) and invasive SCCs, derived from a total of 60 cases of cervical biopsies and cervical conizations, has revealed an increased nuclear translocation of both p-mTOR^(Ser2448)^ and p70S6K^(Thr389)^, indicating the constitutive activation and overexpression of the mTOR pathway in HSIL and SCC [82]. All the studies show that mTOR activation occurs in at least 60% of the HPV-caused cancer patients, consistent with the Akt activation data discussed above, suggesting that mTOR activation may play an important role in most of the HPV-induced carcinogenesis.

mTOR is a crucial metabolic sensor in the growth factor receptor (GFR) pathway, which integrates growth factor signals in cells. The increased nuclear translocation of p-mTOR^(Ser2448)^ and p70S6K^(Thr389)^ correlates with overexpression of the upstream signal transducer EGFR, increased cell cycles and mitotic indices [82]. The activated PI3K/ Akt /mTOR signalling pathway induces phosphorylation of the mTOR complex 1 substrates ,4E-BP1 and S6K, which leads to induction of the functional protein translational machinery and inhibition of autophagy at the early stages of virus-host cell interaction [80]. All these events are partially dependent upon activation of EGFR. Preclinical studies have shown that both the mTOR inhibitor (rapamycin) and EGFR-tyrosine kinase inhibitor (erlotinib) can induce growth delay of xenografted HPV-containing cervical carcinoma cells [83].

A high level of p-mTOR can serve as an independent prognostic marker to predict poor response to chemotherapy and survival of cervical cancer patients [63]. Concurrent use of mTOR inhibitors such as rapamycin and RAD001 and standard-of-care cisplatin/radiation therapy (CRT) has been applied in HPV(+) HNSCC and CCSCC tumour xenografts and mouse models for evaluating the preclinical efficacy of mTOR inhibition [77,84]. Both inhibitors effectively decrease mTOR activity, leading to a remarkable decrease in tumor burden [77] and prolonged survival in immunocompromised mice [84]. It has also been reported that treatment with PI3K inhibitors combined with NaBT significantly decreases the viability of cervical cancer HeLa cells. Inhibition of PI3K enhances NaBT-mediated apoptosis through activation of caspase 3 and caspase 9 and the cleavage of poly (ADP-ribose) polymerase (PARP) [85]. Taken together, these studies provide a rationale for the clinical application of PI3K/mTOR inhibitors as a molecular targeted approach for treating HPV-associated cancers.

## HPV oncogene-mediated PI3K/Akt/mTOR signalling pathway

Recently, several review papers have provided comprehensive summaries of the biological and biochemical activities of three HPV oncoproteins: E6, E7 and E5 [86-88]. Here, we focus on discussing the oncogenic activities of HPV E6, E7 and E5 proteins in inducing the PI3K/Akt/mTOR signalling pathway (Figure 1). Human keratinocytes, a special type of epithelial cells that have a finite life span and do not undergo spontaneous immortalization, are the host cells of HPV infection, [89]. Following HPV infection, the keratinocytes are immortalized and transformed by the viral oncogenes (E6/E7) that act on multiple cellular events including inhibition of p53 and pRb [90-92], altered expression of multiple genes (approximately 4% of the genes on the array) [26] and activation of several signalling pathways, especially, the PI3K/Akt/mTOR signalling pathway [89,93-95]. The PI3K/Akt/mTOR pathway may in turn mediate multiple cellular functions necessary for HPV-induced carcinogenesis (Figure 1) [96-98].

### E6 oncogene

HPV E6 oncoproteins are the key players in HPV-induced cancers. The E6 oncoproteins from high-risk mucosotrophic HPVs (α-HPVs) target not only P53, but also a range of host-cell proteins for proteasome-mediated degradation, resulting in alteration of multiple cellular and molecular events [99-101]. A genome-wide analysis has shown that E6 up-regulates many genes at the transcript level associated with cancer hallmarks including cell cycle, migration, PI3K/Akt /mTOR signalling to mediate cellular transformation [102]. The high-risk HPV E6 oncoproteins contain a PDZ-binding domain; a common structural domain of 80–90 amino acids found in the signalling proteins of multiple organisms [103]. The PDZ-binding domain plays a key role in HPV-mediated cellular transformation. Through this domain, the E6 targets a member of the group of PDZ domain-containing molecules that are mediated by the PI3K/Akt signals [98,102,104]. For example, HPV 16/18 E6 proteins promote proteasome-mediated degradation of human disc large (hDlg) tumor suppressor protein by binding to the second PDZ domain of the hDlg through their C-terminal xS/TxV/L (where x represents any amino acid, S/T serine or threonine, and V/L valine or leucine) motif [2,105]. High-risk HPV E6 oncoproteins efficiently degrade members of the PDZ domain-containing membrane-associated guanylate kinase (MAGUK) family and a PDZ protein, Na (+)/H (+) exchange regulatory factor 1 (NHERF-1) [103]. E6 degrades MAGUK by binding to it with inverted domain structure 1 (MAGI-1), which is one of the most strongly bound PDZ domain-containing substrates of E6. E6 interacts with MAGI-1 to facilitate the perturbation of tight junctions. Restoration of MAGI-1 expression in HPV positive tumour cells induces cell growth arrest and apoptosis [106].

HPV E6 variants (E6*) can act as an adaptor molecule linking a ubiquitin ligase to target proteins, which contain class 1 PDZ domains and are involved in cell junction stability and signalling [100]. E6* proteins differentially modulate hDlg degradation to rebound the levels of activated PTEN and Akt and strongly enhance expression of p-PI3K contributing to activate MAPKs and promote cell proliferation [2,102]. High-risk HPV E6 can target certain substrates both directly and indirectly through the E6* proteins and the two E6 proteins may cooperate in their degradation [100]. In the absence of full-length HPV-18 E6, HPV-18 E6* expression also downregulates the expression levels of Akt, Dlg, and Scribble [100]. It has also been reported that HPV16 E6 and HPV18 E6* oncoproteins activate MAPK signalling pathway to promote cell proliferation by upregulating p-PI3K [102,107]. HPV18 intra-type variations may result in differential abilities to activate cell-signalling molecules such as Akt and MAPKs, directly involved in cell survival and proliferation [102]. Functional studies confirm that HPV18 E6 from an African variant has a major effect on the cellular processes including cell cycle and migration [108]. A specific E6 (amino acid 83) (E6^aa83V^) variant is also linked to invasive tumours. The E6^aa83V^ variant activates PI3K signalling pathway and strengthens the possibility of the existence of Ras-independent mechanisms to recreate signalling through classical Ras effector pathways [107]. The variant also enhances MAPK signalling and cooperative transformation with deregulated Notch1 signalling. These studies suggest that intra-type genome variations of high risk HPVs may differ in their abilities to mediate Akt /MAPKs signalling, thus presenting a differential threat to the development of cervical and other cancers.

E6 proteins of three HPVs (HPV1, 8 and 16), and BPV1 interact with acidic LxxLL motifs of transcriptional coregulator MAML1 to target many host proteins such as the mammalian target of rapamycin complex 1 (mTORC1) to delay keratinocyte differentiation [109-111]. The interaction of HPV-8 E6 with MAML1 causes delay of keratinocyte differentiation [111]. According to the crystal structure analysis, both BPV1 and HPV16 E6 proteins contain two zinc-finger domains and a linker helix [109,110]. Both E6 proteins can bind to LxxLL motifs of the focal adhesion protein paxillin and the ubiquitin ligase E6AP, respectively to form a basic-hydrophobic pocket. The basic-hydrophobic pocket captures the helical LxxLL motifs to stimulate mTORC1 signalling, and cap-dependent translation, through activation of the PDK1 and mTORC2 kinases leading to genetic alterations [109,110]. Such genetic alterations include intra-type genome variations of the virus and changes in chromatin proteins and histone modifications in host cells during HPV16-induced carcinogenesis [107]. The integrity of LxxLL and PDZ protein binding domains is important for activation of cap-dependent translation by high-risk mucosal HPV E6 proteins [109,110].

Generally, β-HPV E6 proteins interact with fewer cellular proteins as is also observed for the α-HPV E6 [58]. This is because β-HPVs such as HPV5 and HPV8 E6 proteins lack the domains for binding to the LxxLL and PDZ motifs. An exception is that both α- and β-HPV E6 proteins can directly interact with p300 protein, a transcriptional co-activator. The interaction appears to be much stronger with β-HPV 5/8 E6 than with α-HPV 16 E6 or β-HPV 38 E6 [58]. Enhanced interaction between β-HPV 5/8 E6 and p300 leads to p300 degradation and the blockage of Akt/p300 association in a proteasomal-dependent but E6AP-independent manner [58]. Decreased p300 concomitantly affects downstream signalling events including expression of differentiation markers K1/10 and involucrin. These results reveal a unique way in which β-HPV E6 proteins are able to affect host-cell signaling in a manner distinct from that of the α-HPVs. Furthermore, HPV16 E6 degrade tuberin, the product of mTOR inhibitor tuberous sclerosis complex 2 (e.g., tumour suppressor gene TSC2), by binding to the DILG motif and ELVG motif located in the carboxyl-terminal of Tuberin, which leads to the phosphorylation of p70 S6 kinase (S6K) [112-114]. The E6 binding domain interacting with tuberin is different to that of p53 [113]. The S6K phosphorylation is tightly associated with HPV16 infection in cervical and oesophageal cancers [112]. Immunohistochemical analysis of p-S6K^(Thr389)^ and p-S6^(Ser235/236)^ in 140 cervical cancer and 161 oesophageal cancer specimens has revealed that both p-S6K and p-S6 were detected significantly more frequently in the HPV16-infected cervical cancer specimens than those in the HPV16-negative specimens [112]. HPV16 E6 activates S6K via Akt signalling, which promotes S6K phosphorylation and sustains the activity of the mTORC1 and mTORC2 signalling cascade [112,115]. Alternatively, HPV16 E6 increases the mTORC1 activity through enhanced phosphorylation of mTOR and activation of the downstream signalling through S6K and eukaryotic initiation factor binding protein 1 (4E-BP1) [116]. HPV16 E6 also causes Akt activation through the upstream kinases PDK1 and mTORC2 under conditions of nutrient deprivation. HPV16 E6 increases protein synthesis by enhancing translation initiation complex assembly at the 5′ mRNA cap. The increase in cap-dependent translation likely results from HPV16 E6-induced Akt /mTORC1 activation, as the assembly of the translation initiation complex and cap-dependent translation are rapamycin sensitive. HPV16 E6-mediated activation of mTORC1 signalling and cap-dependent translation may be a mechanism employed by HPV to promote viral replication in HPV oncoprotein-expressing proliferating cells under conditions of limited nutrient supply [116].

NHERF-1 is a molecular pathway organizer that plays an important role in a number of cellular processes including signal transduction, cellular transformation and recruitment of membrane, cytoplasmic, and cytoskeletal signalling proteins into functional complexes [117]. HPV16 E6 mediated-NHERF-1 degradation correlates with the activation of the PI3K/Akt pathway during carcinogenesis [103]. HPV16 E7 plays a concerted role in E6 mediated NHERF1 degradation [103]. E7 activates the cyclin-dependent kinase complexes to promote the accumulation of a phosphorylated form of NHERF-1 that is preferentially targeted by E6. However, HPV18 E6 does not degrade NHERF-1, suggesting that HPV E6-induced NHERF-1 degradation is HPV type-dependent [103]. In addition, E6-upregulated cIAP2 protein confers resistance to cisplatin in HPV 16/18-infected lung cancer through EGFR/PI3K/Akt pathway [118]. Thus, EGFR or PI3K inhibitor combined with cisplatin may improve the chemotherapeutic efficacy in HPV-induced cancers [118].

### E7 oncogene

HPV E7 protein is responsible for pRb disruption in HPV-induced carcinogenesis. E7 binds to and inactivates pRb to disturb the normal cell division process, allowing the cells to grow out of control and unhindered and thus become cancerous. Clinically, decreased Rb expression is consistently associated with increased CIN grade in the HPV-infected woman’s cervices. It has been reported that HPV E7 significantly up-regulates Akt activity in differentiated keratinocytes, which depends on the ability of E7 binding to and inactivating the proteins of pRb family [17] . Up-regulation of AKT activity and loss of pRb were observed in HPV-positive cervical high-grade squamous intraepithelial lesions when compared with normal cervical tissue. Therefore, pRb expression is inversely correlated with Akt activity in HPV-positive cervical high-grade squamous intraepithelial lesions [17]. E7 directly activates Akt by phosphorylating it at two key sites (threonine 308 and serine 473), which subsequently leads to phosphorylation of BAD, a downstream target of Akt [16]. Akt phosphorylation is associated with activated Notch1 signalling that regulates the PI3K pathway [27,49]. It has been reported that protein phosphatase 2 (PP2 or PP2A), a ubiquitous and conserved serine/threonine phosphatase, interacts with the 35 kDa catalytic and 65 kDa structural subunits of p-Akt to dephosphorylate Akt [119]. Akt dephosphorylation results in loss of its activity in preventing cell apoptosis. HPV E7 binds to the two PP2A subunits to prevent their interactions with p-Akt, thereby maintaining Akt signal activation [16].

Through the PI3K/Akt signalling pathway, HPV E7 oncoprotein inhibits the functions of two cyclin-dependent kinase inhibitors, p21^Cip1^ and p27^Kip1^ [120,121]. As a tumour suppressor, p21^Cip1^ binds to the cyclin E/CDK2 complex to maintain Rb in a phosphorylated state [76]. In the absence of immortalizing oncogenes or genetic lesions, activation of the Raf/Ras pathway results in a p21^Cip1^-dependent cell cycle arrest [122]. In contrast, in the E7-transformed human primary cells, E7 cooperates with Ras to abolish the p21^Cip1^-mediated growth arrest [121]. E7 bypasses Raf-induced arrest and alleviates inhibition of cyclin E-CDK2 without suppressing Raf-specific synthesis of p21^Cip1^ or derepressing p21^Cip1^-associated CDK2 complexes by sustaining Akt activity [2,123,124]. P27Kip1 is a marker of poor prognosis in several forms of cancer when localized to the cytoplasm and has been implicated as a positive regulator of cellular motility [120]. HPV 16 E7 protein can modulate the cytoplasmic localization of p27^Kip1^ and may in turn regulate tumor metastasis/aggressiveness through the PI3K/ Akt pathway [120]. E7 also antagonizes the ability of p27^Kip1^ to block cyclin E-associated kinase and to inhibit transcription of cyclin A *in vitro* [125].

Apoptosis as a normal process of cellular self-destruction or suicide is one of the major contributors to the development of a normal immune system, which serves a protective role in our bodies. In response to oncogenic insults, normal human cells execute a defence response that culminates in apoptosis [126]. In HPV infection, expression of E6/E7 oncogens induces cellular immortalization and transformation and carcinogenesis through the immune evasion or resistance against apoptosis and adaptive immune surveillance*.* Several studies have reported that activation of Akt induced by HPV E7 expression plays a crucial role in immune resistance [126-128]. Due to HPV16 E7 expression, activation of Akt in TC-1/PO and A17 tumours induces an immune resistance against apoptotic cell death [127]. The E7-induced activation of Akt in A17 tumor cells also contributes to significantly upregulate expression of the key antiapoptotic proteins including Bcl-2, Bcl-xL, phosporylated Bad (p-Bad), Bcl-w, cIAP-2 and surviving [127]. Treatment of A17 tumor cells with the Akt inhibitor, API-2, reduces the expression of the antiapoptotic proteins markedly leading to an increase in the apoptosis of tumor cells [128]. It has also been reported that overexpression of E6/E7 from the high-risk HPV16 significantly upregulates expression of cellular inhibitor of apoptosis protein 2 (c-IAP2), which is necessary for the E6/E7-induced resistance to apoptosis and cell survival in HPV16 E6/E7-immortalized human oral keratinocytes [128]. Akt inhibitors markedly abrogate the antiapoptotic effect of c-IAP2 and some other antiapoptotic proteins on different cancer cells [129,130].

Furthermore, normal human diploid fibroblasts expressing the HPV16 E7 oncoprotein are predisposed to apoptosis when they are deprived of growth factors such as IGF-1 in serum-starved medium [126]. The apoptosis of serum-starved HPV16 E7-expressing cells is directly associated with low phosphorylation of Akt and highly activated caspase 3 that plays a central role in the execution-phase of cell apoptosis. Exogenously added IGF-1 can partially inhibit the cell death response associated with upregulated p-Akt in serum-starved E7-expressing cells [126]. In support of these previous findings, we observed that HPV16 E7 inhibits IFN-γ-mediated MHC class I antigen presentation and CTL-induced lysis through blocking interferon regulatory factor-1 (IRF-1) expression in mouse keratinocytes [131]. IRF-1 is a tumor suppressor that can regulate gene expression involved in induction of apoptosis and cell growth control by reducing p-Akt expression [132]. Thus, the activation of PI3K/Akt pathway induced by HPV E6/E7 oncogenes may represent a new mechanism of immune escape and have important implications for developing a novel strategy in cancer immunotherapy against immune-resistant tumor cells [127,128].

As mentioned above, keratinocytes are the host cells of HPV infection. In normal epithelial tissues, cell division and proliferation of keratinocytes are confined to the basal layer, where mitogenic signals are balanced by survival signals transmitted through PI3K/Akt pathway [133]. Once in the suprabasal layer, keratinocytes stop dividing and enter a differentiation program. Primary keratinocytes in *in vitro* cultures resemble *in vivo* epidermal development when they enter a differentiation program [134]. We and others have previously observed that both human and mouse primary keratinocytes grown *in vitro* proceed to cell differentiation with downregulation of proliferation markers including K14 and K5 and upregulation of differentiation markers such as involucrin and K10 [36,135,136]. However, expression of HPV 16 E7 in human foreskin keratinocytes in *in vitro* cultures induces phosphorylation of AKT on threonine 308 and serine 473 to significantly inhibit cell differentiation and cause hyperproliferation [16,17]. It has been reported that a dual epidermal growth factor receptor (EGFR) and HER2 inhibitor Lapatinib reduces expression of E6/E7 and Akt phosphorylation to prevent cell proliferation and induce cell death in HPV-positive cell lines [137]. The HPV E7-activated Akt also enhances keratinocyte migration through downregulation of RhoA activity [120]. Either treatment of PI3K or AKT inhibitors or PIK3CA siRNA transfection results in a significant decrease of E7 expression and E7-induced Akt phosphorylation, consequently, leading to that cellular viability and migration are dramatically reduced in HPV16-transfected keratinocytes [65]. The HPV E7-activated Akt regulates not only tumourigenesis and invasion [138], but also tumor metastasis/aggressiveness by modulating the cytoplasmic localization of p27 [120].

### E5 oncogene

HPV E5 gene encodes an 83-amino acid, membrane-bound protein, which plays an important role in early cervical carcinogenesis by regulating several cellular pathways [139-141]. HPV16 E5 itself cannot immortalize human or mouse primary cells, but can enhance the immortalization of keratinocytes by E6/E7[142] and potentiate the transforming activity of E7 in murine fibroblasts and activation of EGFR in human keratinocytes that naturally express high levels of EGFR after EGF stimulation [104,143-146].

HPV16 E5 induces the anchorage-independent growth of murine fibroblasts by overexpressing EGFR [147]. HPV16 E5 also induces expression of VEGF, which plays a central role in switching on angiogenesis during early cervical carcinogenesis through activation of EGFR and phosphorylation of Akt and ERK1/2 [148,149]. Thus, HPV 16 E5 may activate the EGFR/PI3K/Akt/MEK/ERK1/2 pathway. Recently, it has been reported that expression of HPV16 E5 in undifferentiated keratinocytes alters the key paracrine mediator of epithelial homeostasis, keratinocyte growth factor receptor (KGFR/FGFR2b) [146]. KGFR down-modulation, together with a ligand-dependent decrease of p63, is responsible for a E5-mediated decrease of the early differentiation marker K1 and impairment of keratinocyte differentiation [146].

HPV E5 may act as a survival factor as the E5-expressing cells in human keratinocyte culture exhibit a significant reduction in UVB-irradiation induced apoptosis [145]. A genome-wide microarray assay reveals that E5 expression significantly alters expression of 179 genes including upregulation of PI3K and PKCδ and downregulation of lamin A/C protein, which lead to inhibition of apoptosis and the establishment of persistent infection in the epithelium [150]. The E5-mediated protection against apoptosis can be blocked by two specific inhibitors of the PI3K/MAPK pathways (wortmannin and PD98059), suggesting that the PI3K/MAPK pathways are involved in the protection from apoptosis by HPV16 E5 [145]. Inhibition of the PI3K/Akt signalling prevents the down-regulation of KGFR/p63, supporting an oncogenic role of E5 through the PI3K/Akt pathway [146]. In addition, two BPV1 E5 mutants are severely defective for focus formation, but still competent for enhanced growth through the PI3K/Akt/cyclin D3 pathway together with a Grb2-Gab1-SHP2 complex and JNK protein [151,152]. Thus, it appears that HPV E5 oncoprotein can directly or indirectly target several other substrates to regulate the PI3K/Akt /mTOR pathway.

## HPV pseudovirions and PI3K/Akt /mTOR signals

**Figure 2:**
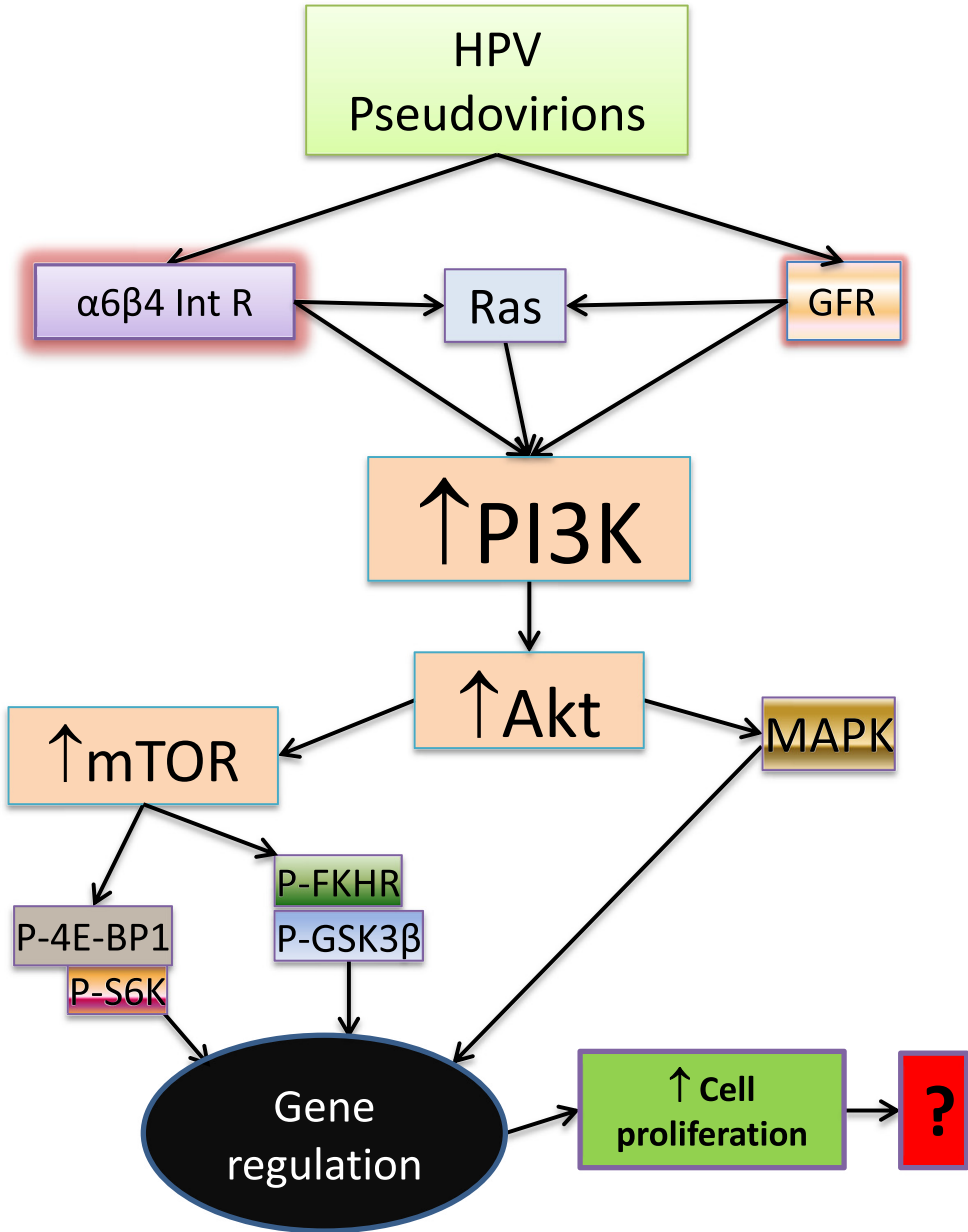
HPV pseudovirions enter the host epithelial cells (keratinocytes) by interacting with α6β4 integrin receptor [154] or growth factor receptor [80] to enhance cell proliferation through the activation of the PI3K/Akt/mTOR and PI3k signalling pathway. Without the oncogenic roles of E6/E7E5, it is impossible for HPV pseudovirions to induce formation of cancerous cells by activating the PI3K/Akt signalling pathway.

It is well documented that induction and progression of tumours by HPV infection are driven by the continuing expression of E6 and E7 oncogenes that degrade and inactivate p53 and pRb, respectively [122,153]. However, two studies have reported that HPV pseudovirions and virus-like particles (VLPs, or as pseudovirions), which do not contain E6/E7 genes or their protein products, also can activate PI3K signalling in human keratinocytes and epidermoid carcinoma cells through the signals of growth factor receptor (GFR) [80] and α6β4 integrin receptor [154]. The pseudovirions-induced PI3K activity results in efficient activation of its two down streamers Akt and mTOR and subsequent phosphorylation of the mTOR complex 1 substrates 4E-BP1 and S6K [80] and of FKHR and GSK3β (Figure 2) [154]. These events combined with activation of Ras/MAPK to enhance cell proliferation and inhibit autophagy [80,154].

Generally, it is impossible that attachment of HPV pseudovirions and other viruses to the surface of cells activates the PI3K/Akt signalling pathway leading to the development of cancerous cells. However, several other published studies appear to suggest that PI3K signalling pathway plays a critical role in cellular entry of pseudovirions of HPV [155] and other viruses such as Zaire Ebola virus (ZEBOV) [156], SV 40 [157] and Epstein–Barr virus (EBV) [158]. Infection with either radiation-inactivated ZEBOV virus or SV40 VLPs activates PI3K/Akt by expression of phosphorylated PI3K/Akt in the infected cells [157,158]. On the other hand, inhibition of PI3K significantly reduces infection rate of HPV 16 pseudovirions (50–60% reduction) [155] and ZEBOV VLPs [156]. In addition, EBV latent membrane protein 2A can transform the EBV-infected cells to become cancerous through constitutive activation of the PI3K/Akt/Ras/MAPK pathway [158]*.* Nontheless*,* whether the PI3K/Akt pathway can play a role in HPV-induced carcinogenesis independent of E6/E7 proteins remains questioned. Thus, more detailed studies are required to improve our current understanding of the full spectrum of mechanisms underlying HPV-induced carcinogenesis.

## Concluding remarks

Recently, the PI3K/Akt/mTOR pathway has been identified as an important signalling pathway to tightly modulate many cellular events including the physiological activities of mitogenic or oncogenic factors, leading to the genesis of many human cancers. Published studies have shown that expression of HPV E6/E7 oncoproteins induces HPV transformed cells to be cancerous not only causing degradation and destabilization of p53 and pRb, but also altering multiple cellular and molecular events through activation of the PI3K/Akt/mTOR signalling pathway. The PI3K/Akt/mTOR signalling pathway in HPV-infected cells is activated through both mutation of the pathway components and activation of upstream signalling molecules. Activation of this pathway contributes to genetic instability, deregulation of proliferation, resistance to apoptosis, and changes in metabolism characteristics, eventually leading to the malignant transformation of the infected cells. This signalling pathway may potentially represents both a great therapeutic opportunity and a practical challenge for treating HPV-induced cancers. Thus, further understanding of the molecular mechanisms by which HPV infection activates the PI3K/Akt/mTOR signalling pathway and the biological roles of this pathway in HPV-induced carcinogenesis will improve the disease prevention, patient care, and surveillance strategies for HPV-positive cancers. We suggest that one important research direction will be to devise the novel biomarker-driven therapeutic strategies to target the PI3K/Akt/mTOR pathway in HPV-associated cancers with a specific molecular profile and evaluate the efficacy of the potential therapeutic agents.

## Abbreviations

ASCCs: Anal squamous cell carcinomas
AIN: Anal intraepithelial neoplasia
ASF/SF2: Alternative splicing factor/splicing factor 2
BCDD: Butyl 2-cyano-3, 11-dioxours-1,12-dien-24-oate
CDK: Cyclin-dependent kinase
CIN: Cervical intraepithelial neoplasia
4E-BP1: Eukaryotic initiation factor binding protein 1
EGFR: Epithelial growth factor receptor
ERK1/2: Extracellular signal-regulated kinase-1/2
FAK: Focal adhesion kinase
GFR: Growth factor receptor
GSK3β: Glycogen synthase kinase-3 beta
HCC: Human cervical carcinoma tissues
hDlg: Human disc large
HFKs: Human foreskin keratinocytes
HIF-1α: Hypoxia-inducible factor-1alpha
HMECs: Human mammary epithelial cells
HPV: Human papillomavirus
HR: High-risk
HSIL: High grade cervical squamous intraepithelial lesion
IGF-1: Insulin-like growth factor 1
JNK: c-Jun N-terminal kinase
KCC1: KC1 co-transport-1
MAPK: Mitogen-activated protein kinase
MAGUK: Membrane-associated guanylate kinase
mTOR: Mammalian target of rapamycin
c-iNOS: Inducible nitric oxide synthases
NHERF-1: Na(+)/H(+) exchange regulatory factor 1
PI3K: Phosphatidylinositol 3-kinases
PIN: Penile intraepithelial neoplasia
PTEN: Phosphatase and tensin homolog
Rb: Retinoblastoma
S6K: S6 kinase
SCC: Squamous cell carcinoma
SDF-1: Stromal Derived Factor-1
SEK-1: Extracellular signal-regulated kinase 1
Skp2: S phase kinase-associated protein 2
SOS-1: Son of seven less homolog 1
TSC2: Tuberous sclerosis complex 2
VEGF: Vascular endothelial growth factor
VIN: Vular intraepithelial neoplasia
VLPs: Virus-like particles
